# Supplementary material for: The step-wise pathway of septin hetero-octamer assembly in budding yeast
Source: eLife. 2017 May 25;6:e23689. doi: 10.7554/eLife.23689 (PMC5461111; doi:10.7554/eLife.23689)
Supplement: Supplementary file 1. — Table listing the strains and plasmids used in this study, including genotypes and origins. DOI: http://dx.doi.org/10.7554/eLife.23689.021 [file elife-23689-supp1.docx]

**Yeast strains**

| **Strain** | | **Genotype** | | **Source** |
| --- | --- | --- | --- | --- |
| BY4741 | | MATa *his3∆1 leu2∆0 ura3∆0 met25∆0* *SUC2 gal2 mal2 mel flo1 flo8-1 hap1 ho bio1 bio6* | | (Brachmann et al., 1998) |
| BY4742 | | MATα *his3∆1 leu2∆0 ura3∆0 lys2∆0* *SUC2 gal2 mal2 mel flo1 flo8-1 hap1 ho bio1 bio6* | | (Brachmann et al., 1998) |
| BY4743 | | MATa/MATα *his3Δ1/his3Δ1 leu2Δ0/leu2Δ0 ura3Δ0/ura3Δ0 lys2Δ0/+ met25Δ0/+ SUC2 gal2 mal2 mel flo1 flo8-1 hap1 ho bio1 bio6* | | (Brachmann et al., 1998) |
| YEF473 | | *his3 leu2 lys2 trp1 ura3* | | (Bi and Pringle, 1996) |
| S288C | | *SUC2 gal2 mal2 mel flo1 flo8-1 hap1 ho bio1 bio6* | | (Mortimer and Johnston, 1986) |
| YO802 | | YEF473-derived: MATα *CDC12-V_C_::KanMX6* | | (Oh et al., 2013) |
| YO685 | | YEF473-derived: MATα *CDC3-V_C_::HIS3MX6* | | (Oh et al., 2013) |
| YO1057 | | YEF473-derived: MATα *CDC10-V_C_::TRP1* | | (Oh et al., 2013) |
| MMY0266 | | BY4741-derived: MATα *cdc10(D182N)-V_C_::KanMX6* | | This study |
| YEF5691 | | YEF473-derived: MATa *CDC11-V_N_::TRP1* | | (Oh et al., 2013) |
| YEF5693 | | YEF473-derived: MATa *SHS1-V_N_::TRP1* | | (Oh et al., 2013) |
| YEF5692 | | YEF473-derived: MATa *CDC12-V_N_::TRP1* | | (Oh et al., 2013) |
| YEF5689 | | YEF473-derived: MATa *CDC3-V_N_::TRP1* | | (Oh et al., 2013) |
| MMY0156 ^b^ | | BY4741-derived: MATa *CDC11-C_N_::KlURA3* | | This study |
| MMY0155^b^ | | BY4741-derived: MATa *SHS1-C_N_::KlURA3* | | This study |
| MMY0191 ^b^ | | BY4741-derived: MATa *CDC10-C_N_::KlURA* | | This study |
| 12-V_C_/11-C_N_^c^ | | MATa/MATα YO802 x MMY0156 diploid | | This study |
| 12-V_C_/S-C_N_^c^ | | MATa/MATα YO802 x MMY0155 diploid | | This study |
| 12-V_C_/3-V_N_^c^ | | MATa/MATα YO802 x YEF5689 diploid | | This study |
| 3-V_C_/12-V_N_^c^ | | MATa/MATα YO685 x YEF5692 diploid | | This study |
| 3-V_C_/10-C_N_^c^ | | MATa/MATα YO685 x MMY0191 diploid | | This study |
| 10-V_C_/3-V_N_^c^ | | MATa/MATα YO1057 x YEF5689 diploid | | This study |
| 10-V_C_/10-C_N_^c^ | | MATa/MATα YO1057 x MMY0191 diploid | | This study |
| 10(D182N)-V_C_/3-V_N_^c^ | | MATa/MATα MMY0266 x YEF5692 diploid | | This study |
| 10(D182N)/10-C_N_^c^ | | MATa/MATα MMY0266 x MMY0191 diploid | | This study |
| 12-V_N_/10-C_N_ ^c^ | | MATa/MATα YEF5692 x MMY0191 diploid | | This study |
| 12-V_C_/3-V_N_/11-C_N_ ^c,d^ | | MATa/MATα YO802 x YEF5689 MATα spore mated to MMY0156 | | This study |
| 12-V_C_/3-V_N_/S-C_N_ ^c,d^ | | MATa/MATα YO802 x YEF5689 MATα spore mated to MMY0155 | | This study |
| 3-V_C_/12-V_N_/10-C_N_ ^c,d^ | | MATa/MATα YO685 x YEF5692 MATα spore mated to MMY0191 | | This study |
| 10-V_C_/3-V_N_/10-C_N_ ^c,d^ | | MATa/MATα YO1057 x YEF5689 MATα spore mated to MMY0191 | | This study |
| 10(D182N)-V_C_/3-V_N_/10-C_N_ ^c,d^ | | MMY0266 x YEF5692 spore mated to MMY0191 | | This study |
| JTY5396 | | BY4741-derived: MATa *CDC10-mCherry::KanMX CDC11- GFP::HIS3MX* | | (McMurray et al., 2011) |
| JTY5397 | | BY4741-derived: MATa *CDC10-mCherry::KanMX SHS1-GFP::HISMX6* | | (McMurray et al., 2011) |
| JTY4365^e^ | | BY4741-derived: MATa *cdc10(D182N)* | | This study |
| JTY3631 | | BY4741-derived: MATa *shs1Δ0::kanMX4* | | (McMurray et al., 2011) |
| JPTA1435 | | S288C-derived: MATα *cdc12(G268R)* | | (Weems et al., 2014) |
| JTY5104 | | BY4742-derived: MATα *cdc10∆*::HIS3MX *cdc3∆*::kanMX | | (McMurray et al., 2011) |

^a^ Vc::KanMX6 cassette was PCR-amplified from pFA6a-V_C_-KanMX6 with homology-flanked primers and integrated at the C-terminal of *cdc10(D182N)* in a mating-type-switched JTY4365.

^b^ C_N_::KlURA3 cassettes were PCR-amplified from PCRII-C_N_-KlURA3 with homology-flanked primers and integrated at the C-terminals of the indicated native locus.

^c^ Diploidy confirmed by passage on -Met, -Lys synthetic media.

^d^ V_­C_/V_­N_ spores were confirmed MATα, *met25Δ0*, and *LYS2*^+^, and retained fluorescent signal at bud necks. They were then mated to the indicated MATa C_N_ strains.

^e^ BY4741 was transformed with *Bgl*II-cut pBEG13. Ura+ transformants were streaked for single colonies, then single colonies were spread on 5-FOA and FOA-resistant clones screened for temperature sensitivity.


**Plasmids**

| **Plasmid** | | **Genotype** | | **Source** | |
| --- | --- | --- | --- | --- | --- |
| pFA6a-V_N_-KanMX | | V_N_ KanMX6 | | (Sung and Huh, 2007) | |
| pFA6a-C_N_-KanMX^a^ | | C_N_ KanMX6 | | This study | |
| pCRII-V_N_-KlURA3^b^ | | V_N_ KanMX6 | | This study | |
| PCRII-C_N_-KlURA3^c^ | | C_N_ KlURA3 | | This study | |
| pFA6a-V_C_-KanMX | | V_C_ KanMX6 | | (Sung and Huh, 2007) | |
| pBEG13 | | *cdc10(D182N)* *URA3* | | (Johnson et al., 2015) | |
| pMVB49 | | 2µ *CDC12 LEU2* | | (Versele and Thorner, 2004) | |
| pMVB54 | | 2µ *CDC12(Δ339-407) LEU2* | | (Versele and Thorner, 2004) | |
| pFM650 | | *CEN* *CDC12 LEU2* | | (Sirajuddin et al., 2009) | |
| YCpL-Cdc12(D104A)^d^ | | *CEN CDC12(D104A) LEU2* | | This study | |
| pLA10 | | *CEN CDC10-GFP URA3* | | (Cid et al., 1998) | |
| pCdc10-1-GFP | | *CEN CDC10(D182N)-GFP URA3* | | (McMurray et al., 2011) | |
| pCdc3-GFP | | *CEN CDC3-GFP LEU2* | | (Richman et al., 1999) | |
| YCpL-Cdc3(D289N)-GFP^e^ | | *CEN CDC3(D289N)-GFP LEU2* | | This study | |
| YCpL-Cdc3(Δ1-56)-GFP^f^ | | *CEN CDC3(Δ1-56)-GFP LEU2* | | This study | |
| YCpL-Cdc3(D289N, Δ1-56)-GFP^g^ | | *CEN CDC3(D289N, Δ1-56)-GFP LEU2* | | This study | |
| YCpL-Cdc3(Δ1-100)-GFP^h^ | | *CEN CDC3(Δ1-100)-GFP LEU2* | | This study | |
| YCpL-Cdc3(D289N, Δ1-100)-GFP^i^ | | *CEN CDC3(D289N, Δ1-100)-GFP LEU2* | | This study | |
| pMVB100 | | *CEN CDC3 URA3* | | (Versele and Thorner, 2004) | |
| pFA6a-VC-His3MX6 | | *V_C_ HIS3MX6* | | (Sung and Huh, 2007) | |
| YCpHU-Cdc3-V_C_^j^ | | *CEN CDC3-V_C_ HIS3 URA3* | | This study | |
| YCpHU-Cdc3(Δ1-100)-V_C_^k^ | | *CEN CDC3(Δ1-100)-V_C_ HIS3 URA3* | | This study | |
| pRS316 | | *CEN URA3* | | (Sikorski and Hieter, 1989) | |
| pSB5 | | *CEN CDC11-GFP URA3* | | (Versele and Thorner, 2004) | |
| pRS316-Shs1-GFP^l^ | | *CEN SHS1-GFP URA3* | | This study | |
| pRS314-SHS1-GFP | | *CEN SHS1-GFP TRP1* | | (Mino et al., 1998) | |
| pRS306 | | *URA3* | | (Sikorski and Hieter, 1989) | |
| pMVB39 | | *CEN CDC12 URA3* | | (Versele and Thorner, 2004) | |
| YCpUK-Cdc12-V_C_^m^ | | *CEN CDC12-V_C_ URA3 KanMX6* | | This study | |
| YCpUK-Cdc12(ΔCTE)-V_C_^n^ | | *CEN CDC12(Δ339-407)-V_C_ URA3 KanMX6* | | This study | |
| pFM829 | | *CEN CDC12(T75A) LEU2* | | (Sirajuddin et al., 2009) | |
| pML109 | | *CEN CDC3-GFP LEU2* | | (Nagaraj et al., 2008) | |

^a^ pFA6a-VN-KanMX was mutagenized at the following sites: C139T, G141C, G196A, G197C, A200G, C201G, C205G, T436G, A437C, A440T, and C445G, to convert the N-terminal fragment of Venus to Cerulean.

^b^ PCR product from genomic DNA of Bioneer strain VN_5421 (YDJ1-VN-KlURA3 from Bioneer’s BY4741 V_N_ fusion library) was generated using the universal F2 and R1 CORE primers. 3' A-overhangs were added using Taq pol and dATP, and the resulting product was inserted into pCRII vector, using a TOPO-TA pCRII kit.

^c^ Created by ligating a *Pac*I/*Asc*I-cut F2/KanB PCR product from pFA6a-CN-KanMX (*Dpn*I-treated to destroy KanR/AmpR in the plasmid) into *Pac*I/*Asc*I-cut, FastAP-treated pCRII-VN-KlURA3.

^d^ D104A mutation was introduced to the *CDC12* allele in pFM650 via site-directed mutagenesis.

^e^ D289N mutation was introduced to the *CDC3* allele in pCdc3-GFP via site-directed mutagenesis.

^f^ pCdc3-GFP was linearized via PCR using a primer pair that excluded the codons encoding residues 1-56 of Cdc3. Template plasmids were destroyed with *Dpn*I. PCR product was then converted into functional plasmids via gap-repair using a 70-mer oligo with 35-mer homology to each side of the gap.

^g^ YCpL-Cdc3(D289N)-GFP was linearized via PCR using a primer pair that excluded the codons encoding residues 1-56 of Cdc3. Template plasmids were destroyed with *Dpn*I. PCR product was then converted into functional plasmids via gap-repair using a 70-mer oligo with 35-mer homology to each side of the gap.

^h^ pCdc3-GFP was linearized via PCR using a primer pair that excluded the codons encoding residues 1-100 of Cdc3. Template plasmids were destroyed with *Dpn*I. PCR product was then converted into functional plasmids via gap-repair using a 70-mer oligo with 35-mer homology to each side of the gap.

^i^ YCpL-Cdc3(D289N)-GFP was linearized via PCR using a primer pair that excluded the codons encoding residues 1-100 of Cdc3. Template plasmids were destroyed with *Dpn*I. PCR product was then converted into functional plasmids via gap-repair using a 70-mer oligo with 35-mer homology to each side of the gap.

^j^ pMVB100 was co-transformed into BY4742 with a V_C_-HisMX3 PCR product carrying homologous tails for C-terminal integration into *CDC3*.

^k^ YCpHU-Cdc3-V_C_ was linearized via PCR using a primer pair that excluded the codons encoding residues 1-100 of Cdc3. Template plasmids were destroyed with *Dpn*I. PCR product was then converted into functional plasmids via gap-repair using a 70-mer oligo with 35-mer homology to each side of the gap.

^l^ pRS314-SHS1-GFP was co-transformed into BY4741 with a *URA3* PCR product made with primers 5_pRS and 3_pRS and template pRS306.

^m^ pMVB39 was cut with *Bam*HI and co-transformed into YEF5691 with a PCR product of the V_C_ tag from YO802 using primers with homology to the 3’ end of the *CDC12* coding sequence.

^n^ YCpUK-Cdc12-V_C_ was linearized via PCR using a primer pair that excluded the codons encoding residues 339-407 of the *CDC12* ORF. Template plasmids were destroyed with *Dpn*I. PCR product was then converted into functional plasmids via gap-repair using a 70-mer oligo with 35-mer homology to each side of the gap.

REFERENCES

Bi, E., and Pringle, J.R. (1996). ZDS1 and ZDS2, genes whose products may regulate Cdc42p in Saccharomyces cerevisiae. Mol. Cell. Biol. *16*, 5264–5275.

Brachmann, C.B., Davies, A., Cost, G.J., Caputo, E., Li, J., Hieter, P., and Boeke, J.D. (1998). Designer deletion strains derived from Saccharomyces cerevisiae S288C: a useful set of strains and plasmids for PCR-mediated gene disruption and other applications. Yeast *14*, 115–132.

Cid, V.J., Adamíková, L., Cenamor, R., Molina, M., Sánchez, M., and Nombela, C. (1998). Cell integrity and morphogenesis in a budding yeast septin mutant. Microbiology *144 ( Pt 12)*, 3463–3474.

Johnson, C.R., Weems, A.D., Brewer, J.M., Thorner, J., and McMurray, M.A. (2015). Cytosolic chaperones mediate quality control of higher-order septin assembly in budding yeast. Mol. Biol. Cell.

McMurray, M.A., Bertin, A., Garcia, G., 3rd, Lam, L., Nogales, E., and Thorner, J. (2011). Septin filament formation is essential in budding yeast. Dev. Cell *20*, 540–549.

Mino, A., Tanaka, K., Kamei, T., Umikawa, M., Fujiwara, T., and Takai, Y. (1998). Shs1p: a novel member of septin that interacts with spa2p, involved in polarized growth in saccharomyces cerevisiae. Biochem. Biophys. Res. Commun. *251*, 732–736.

Mortimer, R.K., and Johnston, J.R. (1986). Genealogy of Principal Strains of the Yeast Genetic Stock Center. Genetics *113*, 35–43.

Nagaraj, S., Rajendran, A., Jackson, C.E., and Longtine, M.S. (2008). Role of nucleotide binding in septin-septin interactions and septin localization in Saccharomyces cerevisiae. Mol. Cell. Biol. *28*, 5120–5137.

Oh, Y., Schreiter, J., Nishihama, R., Wloka, C., and Bi, E. (2013). Targeting and functional mechanisms of the cytokinesis-related F-BAR protein Hof1 during the cell cycle. Mol. Biol. Cell *24*, 1305–1320.

Richman, T.J., Sawyer, M.M., and Johnson, D.I. (1999). The Cdc42p GTPase is involved in a G2/M morphogenetic checkpoint regulating the apical-isotropic switch and nuclear division in yeast. J. Biol. Chem. *274*, 16861–16870.

Sikorski, R.S., and Hieter, P. (1989). A system of shuttle vectors and yeast host strains designed for efficient manipulation of DNA in Saccharomyces cerevisiae. Genetics *122*, 19–27.

Sirajuddin, M., Farkasovsky, M., Zent, E., and Wittinghofer, A. (2009). GTP-induced conformational changes in septins and implications for function. Proc. Natl. Acad. Sci. U. S. A. *106*, 16592–16597.

Sung, M.-K., and Huh, W.-K. (2007). Bimolecular fluorescence complementation analysis system for in vivo detection of protein-protein interaction in Saccharomyces cerevisiae. Yeast Chichester Engl. *24*, 767–775.

Versele, M., and Thorner, J. (2004). Septin collar formation in budding yeast requires GTP binding and direct phosphorylation by the PAK, Cla4. J. Cell Biol. *164*, 701–715.

Weems, A.D., Johnson, C.R., Argueso, J.L., and McMurray, M.A. (2014). Higher-Order Septin Assembly Is Driven by GTP-Promoted Conformational Changes: Evidence From Unbiased Mutational Analysis in Saccharomyces cerevisiae. Genetics *196*, 711–727.
